# Cardiovascular Risk Factors and Social Development Index

**DOI:** 10.3389/fcvm.2021.631747

**Published:** 2021-02-23

**Authors:** Mireya Martínez-García, Guadalupe O. Gutiérrez-Esparza, Juan Carlos Roblero-Godinez, Diana Vianey Marín-Pérez, Cindy Lucia Montes-Ruiz, Maite Vallejo, Enrique Hernández-Lemus

**Affiliations:** ^1^Sociomedical Research, National Institute of Cardiology, Mexico City, Mexico; ^2^Cátedras CONACYT, National Council of Science and Technology, Mexico City, Mexico; ^3^Program in Health Promotion, Autonomous University of Mexico City, Mexico City, Mexico; ^4^Program in Nutrition, Juarez Autonomous University of Tabasco, Villahermosa, Mexico; ^5^Program in Nutrition, Juarez University From the State of Durango, Durango, Mexico; ^6^Computational Genomics Division, National Institute of Genomic Medicine, Mexico City, Mexico

**Keywords:** cardiovascular diseases, residence characteristics, cardiovascular risk factors, human development index, social development index, socioeconomic status, social determinants of health

## Abstract

Cardiovascular diseases (CVD) are the leading causes of morbidity and mortality worldwide. The complex etiology of CVD is known to be significantly affected by environmental and social factors. There is, however, a lag in our understanding of how population level components may be related to the onset and severity of CVD, and how some indicators of unsatisfied basic needs might be related to known risk factors. Here, we present a cross-sectional study aimed to analyze the association between cardiovascular risk factors (CVRF) and Social Development Index (SDI) in adult individuals within a metropolitan urban environment. The six components of SDI as well as socioeconomic, anthropometric, clinical, biochemical, and risk behavior parameters were explored within the study population. As a result, several CVRF (waist circumference, waist-to-height ratio, body mass index, systolic blood pressure, glucose, lower high-density lipoprotein cholesterol, triglycerides, and sodium) were found in a higher proportion in the low or very low levels of the SDI, and this pattern occurs more in women than in men. Canonical analysis indicates a correlation between other socioeconomic features and anthropometric, clinical, and biochemical factors (canonical coefficient = 0.8030). Further studies along these lines are needed to fully establish how to insert such associations into the design of health policy and interventions with a view to lessen the burden of cardiovascular diseases, particularly in metropolitan urban environments.

## Introduction

Although much progress has been made in understanding the effect that the complex context of environmental, biological, social, and collective domains has on noncommunicable diseases (NCDs), cardiovascular diseases (CVD) are still the leading causes of death in human populations (1). Most of the burden of CVD has been explained by a set of traditional risk factors that affect both men and women (2). In Mexico, as in other middle-income countries, cardiovascular mortality, as well as, their main traditional and novel risk factors have become a major health problem, causing an economic burden and being an important source of disability in young adults (3–7).

Recently, Mexico is undergoing a transition in the burden of NCDs from groups with high socioeconomic status (SES) to those in low SES that have been systematically neglected (8). Economy and social development have increased, but conversely, healthy food systems and changes in lifestyles have been neglected (9). As a result, the country has undergone a nutrition transition, decreasing the prevalence of malnutrition, whereas the prevalence of obesity has increased in epidemic proportions, especially in women (10).

In the last decades, there has been a national reduction in CVD risk through sophisticated interventions that integrate a vast compendium of information about the populations studied, with promising results (11–13). One of them revealed that knowledge of social and environmental conditions is crucial to elucidate factors that will promote, extend, and increase future adoption of health promotion interventions; especially access to drinking water, fresh food at affordable prices, and safe spaces that facilitate physical activity, some of these are included in the called *Human Development Index (HDI)* (14, 15).

The HDI was introduced by the *United Nations Development Program* (UNDP) in the 1990s to measure the development level inside the regions, but also, to help countries build specific development indices to identify more precisely the social inequalities within them (16, 17). HDI incorporates information on health and education, but does not take into account inequality with the benefits of development that are distributed among the population (18). Different nations have established their own evaluation systems of social development with complex and novel frameworks to assess the well-being of societies and had proposed practical application either in health systems or at the household level (19–22). For example, one study in Germany suggested a *Sustainable Child Development Index (SCDI)* be integrated into databases as *The Social Hot-spots Database* to measure and compare the level of development in different geographical areas, and between population groups (23, 24). Another study suggested the incorporation of the territorial dimension in the research of HDI to limit the territorial inequality that human development presents in Latin American countries, especially regarding health and education (25).

In the particular case of exploring the relationship between cardiovascular risk factors (CVRF) and socioeconomic development using the HDI, some studies have been advancing in relation to coronary heart disease (CHD). Zhu et al. investigated the patterns in Asia, Europe, and North America regions (26). Their result shows that there was a positive association between CHD prevalence and the national HDI in developing countries, whereas in developed countries the association was negative. Also, their global review demonstrated that the positive association of social developmental level and CHD was stronger in males than in females in developing countries, but in developed countries, the females with higher social development levels were less susceptible to CHD (26).

Hence, some aspects of social development (economy, culture, education, healthcare, and environment) might influence the people's capacity to be healthy or diseased, but also, supporting decision makers to develop policies in less favored regions (23, 27). Amartya Sen, Nobel Prize in Economics, points out, *fair and equitable social development can only prosper if at the same time, the investment is made in education, health, and nutrition of human capital* (28).

### Social Development in Mexican Context

Mexico is an upper-middle-income country, is the 13th-largest country in the world, with more than 126 million inhabitants. While Mexico has become a developed country, more than half of their inhabitants lives in a state of precariousness and lack of inclusion conditions and social differences are becoming more acute (29). For decades, social development in health, education, housing, basic services, food, and work, as well as social protection, security, and violence reduction have been unequally distributed in the region (30–32). These circumstances have contributed to modify some health conditions in Mexican men and women with consequences in many periods of life, even beyond future generations (33–36).

Mexico City (CDMX) is the capital of Mexico and one of the 32 states of Federal Entities (37). CDMX is the most populated city in North America, the largest urban agglomeration in the Western Hemisphere, and the second largest in the world (38). In 2016, CDMX concentrated a population near of 9 million people with a land area around of 1,500 km^2^ (37). CDMX is currently splitted into 16 administrative divisions of the city or boroughs (each of them is composed of hundreds of neighborhoods or territorial units) (39).

The *Social Development Index* (SDI) is based on the HDI, and is the instrument used to define, measure, and classify the degree of social development of territorial units. Social development in the Mexican context is a process of economic improvement and equalization of the general welfare conditions, since poverty constitutes the most serious expression of the limitations to improve the quality of life. In this sense, social development is a central objective of the action of the Government of Mexico City. The Political Constitution of the United Mexican States considers as rights for social development, education, health, nutritional, and quality food, housing, the enjoyment of a healthy environment, work, and social security (40).

Also social problems, the major health problems of Mexicans, have been the result of aspects linked to development. IDS surveys, contribute to the generation of information for decision-making in matters of social policy, but also in health care, especially to analyze the inequality of socioeconomic coverage that subsists in the territory of one of the most populated cities in the world (41). Hence, there is a need to study the phenomenon of social development to understand its effects on risk factors for CVD. This study is based on an epidemiological premise that social risks and diseases are not independent, rather there are conditions and determinants that increase or reduce those risks. Therefore, the objective of this study is to analyze the association between Cardiovascular Risk Factors and SDI in Mexican healthy adults.

## Materials and Methods

###  Study Population

This cross-sectional analysis includes the complete data from the baseline visit of 2,084 healthy adults residents of Mexico City, between 20 and 50 years old of *Prospective longitudinal study of risk factors for hypertension incidence in a Mexico City, Tlalpan 2020 cohort* (42). This is the first cohort in Mexico aimed to study the impact of traditional and nontraditional risk factors for systemic hypertension. The study was approved by the Research Ethics Board for Biomedical Research in Humans by the National Institute of Cardiology *Ignacio Chavez* (INC-ICh) under number 13-802. All patients gave written informed consent. The enrollment started in September 2014 and is ongoing. A more detailed description of the study is presented in Supplementary Material.

The participants are being evaluated every 2 years over a period of 10 years or until they develop systemic hypertension (primary outcome). At every visit, the blood pressure, laboratory tests, anthropometric measurements, sociodemographic data, and cardiovascular risk factors are assessed by trained personnel by means of standardized instruments, methods, and structured questionnaire.

###  Sociodemographic Characteristics

Through a personal interview, with a structured questionnaire, the following sociodemographic information is being obtained: marital status (single, married, and other), educational level concluded (elementary school, junior high school, higher, and postgraduate), and occupational class (student, business executive, housekeeper, professional, manually qualifies, manually unqualified, other, and unemployed).

###  Anthropometric and Clinical Parameters

Systolic and diastolic blood pressure are measured according to the *JNC 7* standard procedure; hypertension status (HTN) was defined as a systolic blood pressure ≥ 140 mm Hg and/or a diastolic blood pressure ≥ 90 mm Hg (43). Anthropometric measurements (weight, height, and waist circumference) are being assessed according to *The International Society for the Advancement of Kinanthropometry, ISAK* (44), with standardized instruments such as a mechanical column scale (SECA 700) with capacity of 220 kg and precision of 0.05 kg and a stadiometer SECA 220 (further details can be found in Supplementary Material).

###  Biochemical Test

The blood samples are being obtained after an overnight fast of 12 h at the Central Laboratory of INC-ICh using standardized procedures such as automated photometry, spectrophotometry, potentiometry, and chemiluminescence and were run on the AU 680 Beckman Coulter (2012), Coulter LH Series Pak Reagent Kit. The reference values are as follows: fasting plasma glucose (70–105 mg/dl), triglycerides (40–200 mg/dl), low-density lipoprotein cholesterol (LDL-C) (80–130 mg/dl), high-density lipoprotein cholesterol (HDL-C) (women: >50 mg/dl and men: >40 mg/dl), total cholesterol (140–200 mg/dl), uric acid (women: 3.80–6.20 mg/dl and men: 4.80–8.00 mg/dl), serum creatinine (women: 0.60–1.00 mg/dl and men: 0.70–1.30 mg/dl), Atherogenic Index (LDL-C/HDL-C, elevated defined as a value >4), and serum sodium (136.00–145.00 mmol/l) (45).

A urine of a 24 h collection is being obtained from each participant. Several days before the appointment, precise, and clear indications for the correct urine collection are given to participants (discard the first urine in the morning and collect all urine for a period of 24 h, including the first urine of the following morning, which will be the day of the appointment). Urinary sodium and potassium are determined by the ion selective electrode method, and urinary creatinine is determined by Jaffe's colorimetric assay using an automated analyzer. A urine sample is considered complete when urine creatinine levels are within the standard excretion rate (46). The reference values are as follows: for creatinine in women between 740 and 1,570 mg/24 h and in men between 1,040 and 2,350 mg/24 h, for sodium between 40.00 and 220.00 mmol/24 h and for potassium excretion between 25.00 and 125.00 mmol/24 h. Sodium and potassium excretion was reported in mmol/24 h (or equivalently mEq/24 h) (47).

###  Others Risk Factors

The following traditional and nontraditional CVRF are being explored by validated questionnaires: smoking and alcohol consumption history, physical activity [measured by the long version of *International Physical Activity Questionnaire [IPAQ]: categorized into low, moderate, or high physical activity levels*; (48)], psychological stress level [as determined by the *State-Trait Anxiety Inventory [STAI]* Spanish version; categorized into low, moderate, or severe psychological stress; (49)], and sleep disorders in the last week by means of the Spanish-language Medical Outcomes Study-Sleep scale of 12 items (50). Details of *Tlalpan 2020* study protocol, design, evaluations, and instrumentation have been presented in Supplementary Material.

###  Social Development Index

**Figure 1:**
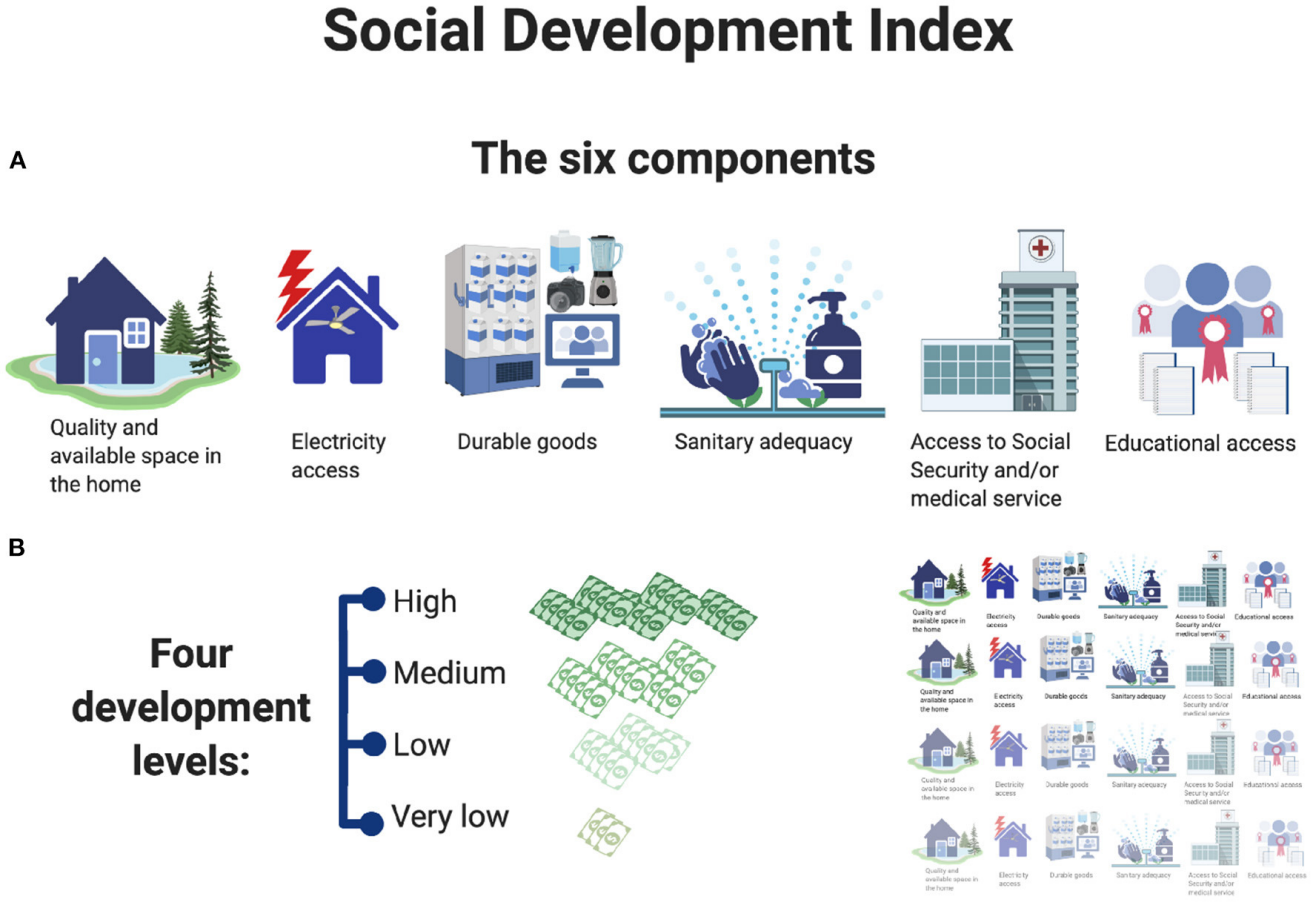
SDI, Social Development Index of Mexico City. **(A)** The six components of Social Development Index and **(B)** the four development levels of Social Development Index.

The SDI in Mexico City is the instrument used to define, measure, and classify the degree of social development of territorial units, geographic spaces that correspond to the subdivision of the municipal geostatistical areas in Mexico City. When calculating the SDI for each territorial unit, the corresponding development layer is obtained as a numerical value between 0.0 and 1.0 that allows to sort the territorial units from worse (less developed) to better (higher social development) (40). According to the value obtained, the socioeconomic conditions are characterized in four development levels: (1) very low, (2) low, (3) medium, and (4) high (51). This SDI has been recommended by economists as a key proxy of household poverty and inequality based on the method of *unsatisfied basic needs* (52). Basic needs are represented through six components which are combined by a weighted arithmetic mean to calculate the poverty intensity (40) (see Figure 1).

The six components of the SDI are as follows:

1. *Quality and available space in the home (QASH)*, which refers to the material of the floor, ventilation, and lighting condition, the number of rooms in the house, and the number of people (occupants) living in it.
2. *Educational access* refers to people 15 years of age or older who reached the basic education level, which in Mexico is high school studies.
3. *Access to social security (ASS)* refers to the right of the household members to any of the Mexican health systems: Mexican Institute of Social Security (Spanish: Instituto Mexicano del Seguro Social, IMSS), Civil Service Social Security and Services Institute (Spanish: Instituto de Seguridad y Servicios Sociales de los Trabajadores del Estado, or ISSSTE), Mexican Petroleum (Spanish: Petroleos Mexicanos, Pemex), Mexican Defense, Mexican Navy, Institute of Health for Well-being (Spanish: Instituto de Salud para el Bienestar, INSABI), or private institutions.
4. *Durable goods* refers to material goods whose value is equal to or greater than (17.81 USD) and/or the possession of at least three material goods (television, gas stove, computer, refrigerator, washing machine).
5. *Sanitary adequacy* is constructed from three sub-components: about the source of water supply, the existence of toilet and access to the drainage system.
6. *Electricity access* is the dichotomous indicator, whether or not it lacks energy adequacy.

###  Statistical Analysis

#### Summary Statistics

Analyses were performed using [R] version 4.0.2 (53). The distribution of numerical data was assessed using the Shapiro–Francia tests and since it was different from the normal standard (*W'* > 0.05); numerical data are presented as median and inter-quartile range (IQR, *Q*1−*Q*3), and qualitative variables as absolute values and percentages (*n*, %).

The levels of the SDI (very-low, low, medium, and high) were compared against the CVRF and also against sexes; therefore, a two-way ANOVA was used for numerical variables. When the interaction term was statistically significant (*p* <0.20), a one way nonparametric test was used to compare the SDI categories within each sex.

For the association among qualitative variables, chi-squared test (χ^2^ at 95% significance) or Cramer's V coefficient (C's V, a value between 0 and 0.2 was considered as no association, and a value between 0.2 and 0.6 as a moderated association and a value larger than 0.6 a strong association) were used when the expected values were equal or <5 (54). The Bonferroni's correction for multiple comparisons was used. A *p-value* ≤ 0.05 was considered statistically significant.

*Alluvial diagram* is a qualitative/semi-quantitative illustration and was employed as a visual representation to highlight and summarize the main relations between cardiovascular risk factors and SDI level by sex. The height of a block represents the size of the cluster and the height of a stream field represents the size of the components contained in both blocks connected by the stream field. Alluvial diagram was made by RAW Graphs, an open source data visualization (55).

#### Multivariate Analysis

Since we wanted to explore the complex simultaneous interactions between two sets of variables (dependent and independent) as well as the linear interrelation between them, canonical correlation was used for the multivariate analysis (56).

The main objective of this type of analysis is to identify the *r* lineal combinations within the first set of variables as follows:

(1) $$\begin{array}{lll} U_{1} & = & a_{11}X_{1}+a_{12}X_{2}+\ldots +a_{1p}X_{p}\\ U_{2} & = & a_{21}X_{1}+a_{22}X_{2}+\ldots +a_{2p}X_{p}\\ \;\vdots & & \vdots \\ U_{r} & = & a_{r1}X_{1}+a_{r2}X_{2}+\ldots +a_{rp}X_{p} \end{array}$$

and within the second set of variables

(2) $$\begin{array}{lll} V_{1} & = & b_{11}Y_{1}+b_{12}Y_{2}+\ldots +b_{1p}Y_{p}\\ V_{2} & = & b_{21}Y_{1}+b_{22}Y_{2}+\ldots +b_{2p}Y_{p}\\ \;\vdots & & \vdots \\ V_{r} & = & b_{r1}Y_{1}+b_{r2}Y_{2}+\ldots +b_{rp}Y_{p} \end{array}$$

The most significant correlation coefficient will be shown between U1 (see Equations 1 and 2) and V1, the second between U2 and V2, and so on. There should be no correlation between U1 and U2 and U3, and neither between V1 and V2 and V3, these combinations are the so-called canonical variables.

For this study, the one set of variables (U) was as follows: DSI, sex, marital status, educational level, and occupational class, and the following set of variables (V) was as follows: biochemical parameters (fasting plasma glucose, uric acid, high-density lipoprotein, low-density lipoprotein, triglycerides, serum creatinine, and urinary potassium and creatinine), systolic blood pressure, waist-to-height-ratio (WHtR), and alcohol consumption.

#### Power Calculations

Statistical power was calculated by F-statistics. For the full study, we have 71 variables so that the maximum possible number of predictors is 70. Hence, the number of degrees of freedom for the numerator *df*_*num*_ = *predictors*−1 = 69. Since we have a sample size of 2,084 individuals, the number of degrees of freedom for the denominator *df*_*num*_ = *samples*−*predictors* = 2014. With these in mind, we obtained a statistical power larger than 99.999% under a significance level of 1*E*−07, even for very mild effect sizes *f*2 = 0.1.

## Results

###  Sociodemographic Characteristics

Data were collected from 2,084 healthy adult residents of Mexico City, 1,314 (63%) women and 770 (37%) men. Table 1 shows the distribution of the SDI and its components among the study population. No meaningful differences were identified; however, interesting contrasts were noted that are worth mentioning. More than 80% of participants were classified as low, medium, or high SDI. In relation to *quality and available space in the home* and *access to social security*, 55 and 95%, respectively, were classified as very low, while more than 90% of participants were classified as medium or high regarding *educational access, durable goods, and sanitary adequacy*. The entire population studied had access to electricity.

**Table 1:** Distribution of Social Development Index (SDI) and its components by level of SDI between women and men.

					***p*-Values**
**Components n(%)**	**Very low**	**Low**	**Medium**	**High**	**χ^2^*or C's V***
**SDI**					
Female	189 (14)	498 (38)	302 (23)	325 (25)	0.882
Male	101 (13)	296 (38)	181 (24)	192 (25)	
**QASH**					
Female	729 (55)	231 (18)	132 (10)	222 (17)	0.994
Male	426 (55)	139 (18)	76 (10)	129 (17)	
**Educational access**					
Female	0	4 (0.3)	171 (13)	1,139 (86.7)	0.028^*^
Male	0	1 (0.1)	89 (11.6)	680 (88.3)	
**ASS**					
Female	1,251 (95)	63 (5)	0	0	0.163
Male	743 (96)	27 (4)	0	0	
**Durable goods**					
Female	10 (1)	36 (3)	441 (33)	827 (63)	0.613
Male	5 (1)	15 (2)	250 (32)	500 (65)	
**Sanitary adequacy**					
Female	160 (12)	174 (13)	274 (21)	706 (54)	0.385
Male	80 (10)	81 (11)	177 (23)	432 (56)	
**Electricity lacking**					
Female	0	0	0	0	**+**
Male	0	0	0	0	

* *p-value of Cramer's V coefficient*.

+ *p-value null: no comparisons were made. QASH, quality and available space in the home; ASS, access to social security*.

Regarding *Marital Status* most participants were married (46%); however, no significant differences were identified in relation to the SDI's levels by sex. *Education level* also showed an interesting distribution among the SDI's levels within each sex. Most women and men, regardless of the SDI level, had an educational level of high school or college; however, it is still perceived that women compared to their male counterparts at lower levels of development have less access to basic education (see Table 2).

**Table 2:** Distribution of Sociodemographic characteristics among levels of Social Development Index (SDI) between women and men.

					***p*****-Values**	
					**χ^2^*or C's V***	**Bonferroni**
**CVRF n(%)**	**Very low**	**Low**	**Medium**	**High**	**uncorrected**	**corrected**
**MARITAL STATUS**						
**Single**						
Female	65 (13)	174 (35)	121 (24)	139 (28)	0.082	0.41^*a*^
Male	37 (12)	111 (37)	74 (24)	83 (27)	0.064	0.32^*b*^
**Married**						
Female	82 (14)	242 (40)	137 (23)	139 (23)	0.965	4.83^*c*^
Male	46 (13)	131 (37)	90 (25)	90 (25)	0.618	3.09^*d*^
**Other**						
Female	42 (20)	82 (38)	44 (20)	47 (22)	0.240	1.20^*e*^
Male	18 (17)	54 (50)	17 (16)	19 (17)		
**EDUCATIONAL LEVEL**						
**Elementary school**						
Female	56 (30)	83 (44)	35 (18)	16 (8)	*p* < 0.001	0^*a*^
Male	32 (24)	60 (45)	25 (19)	16 (12)	*p* < 0.001	0^*b*^
**High school**						
Female	72 (15)	214 (46)	103 (22)	80 (17)	0.591	3.55^*c*^
Male	34 (14)	104 (42)	61 (25)	48 (19)	0.622	3.73^*d*^
**College**						
Female	53 (10)	173 (33)	135 (26)	162 (31)	0.701	4.21^*e*^
Male	30 (9)	117 (37)	74 (23)	97 (31)	0.089^*^	0.53^*f*^
**Postgraduate**						
Female	8 (6)	28 (21)	29 (22)	67 (51)		
Male	5 (7)	15 (21)	21 (29)	31 (43)		

a *Among women*.

b *Among men*.

c *Between women and men in the first category*.

d *Between women and men in the second category*.

e *Between women and men in the third category*.

f *Between women and men in the fourth category*.

* *p-value of Cramer's V coefficient*.

In relation with *Occupational Class*, meaningful differences were observed regarding unemployment status between sexes prior to Bonferroni's correction. In the very low and high SDI's levels, the proportion of women was larger than the men's proportion; in contrast, in the low and medium SDI's levels, the proportion of men was larger than the women's proportion. However, after Bonferroni's correction, the statistical significance disappeared. Other interesting findings were those regarding the housekeeper, an occupational class linked to women; while manually unqualified was linked only to men, these findings suggest gender influences and stereotypes very deeply entrenched in Mexican culture (see Table 3).

**Table 3:** Distribution of Occupational characteristics among levels of Social Development Index (SDI) between women and men.

					***p*****-Values**	
					**χ^2^*or C's V***	**Bonferroni**
**CVRF n(%)**	**Very low**	**Low**	**Medium**	**High**	**Uncorrected**	**Corrected**
**OCCUPATIONAL CLASS**						
**Student**						
Female	20 (14)	47 (33)	32 (23)	42 (30)	0.112	1.01^*a*^
Male	19 (18)	39 (37)	22 (21)	26 (24)	0.160	1.44^*b*^
**Business executive**						
Female	2 (18)	4 (36.5)	4 (36.5)	1 (9)	0.692	6.23^*c*^
Male	0	6 (55)	3 (27)	2 (18)	0.36^*^	3.26^*d*^
**Housekeeper**						
Female	58 (19)	134 (44)	64 (21)	49 (16)	0.064^*^	0.58^*e*^^,^^*f*^
Male	0	1 (100)	0	0	**+**	**+**
**Professional**						
Female	38 (10)	125 (32)	96 (25)	130 (33)	0.666	5.99^*g*^
Male	20 (8)	71 (29)	66 (27)	89 (36)	0.784	7.06^*h*^
**Manually qualify**						
Female	34 (14)	107 (44)	53 (22)	47 (20)	0.914	8.23^*i*^
Male	31 (14)	102 (48)	39 (18)	42 (20)	0.047	0.42^*j*^
**Manually unqualified**						
Female	0	0	0	0		
Male	9 (24)	12 (32)	8 (22)	8 (22)		
**Other occupation**						
Female	17 (18)	33 (34)	22 (23)	24 (25)		
Male	14 (21)	24 (36)	15 (22)	14 (21)		
**Unemployed**						
Female	20 (15)	48 (37)	31 (24)	32 (24)		
Male	8 (9)	41 (47)	28 (32)	11 (12)		

a *Comparison among women*.

b *Comparison among men*.

c *Comparison between women and men in the first category*.

d *Comparison between women and men in the second category*.

e *Comparison between women and men in the third category*.

f *Comparison between women and men in the fourth category*.

g *Comparison between women and men in the fifth category*.

h *Comparison between women and men in the sixth category*.

i *Comparison between women and men in the seventh category*.

j *Comparison between women and men in the tenth category*.

* * Cramer's V coefficient*.

###  Cardiovascular Risk Factors and SDI's Levels

#### Anthropometric and Clinical Parameters

**Figure 2:**
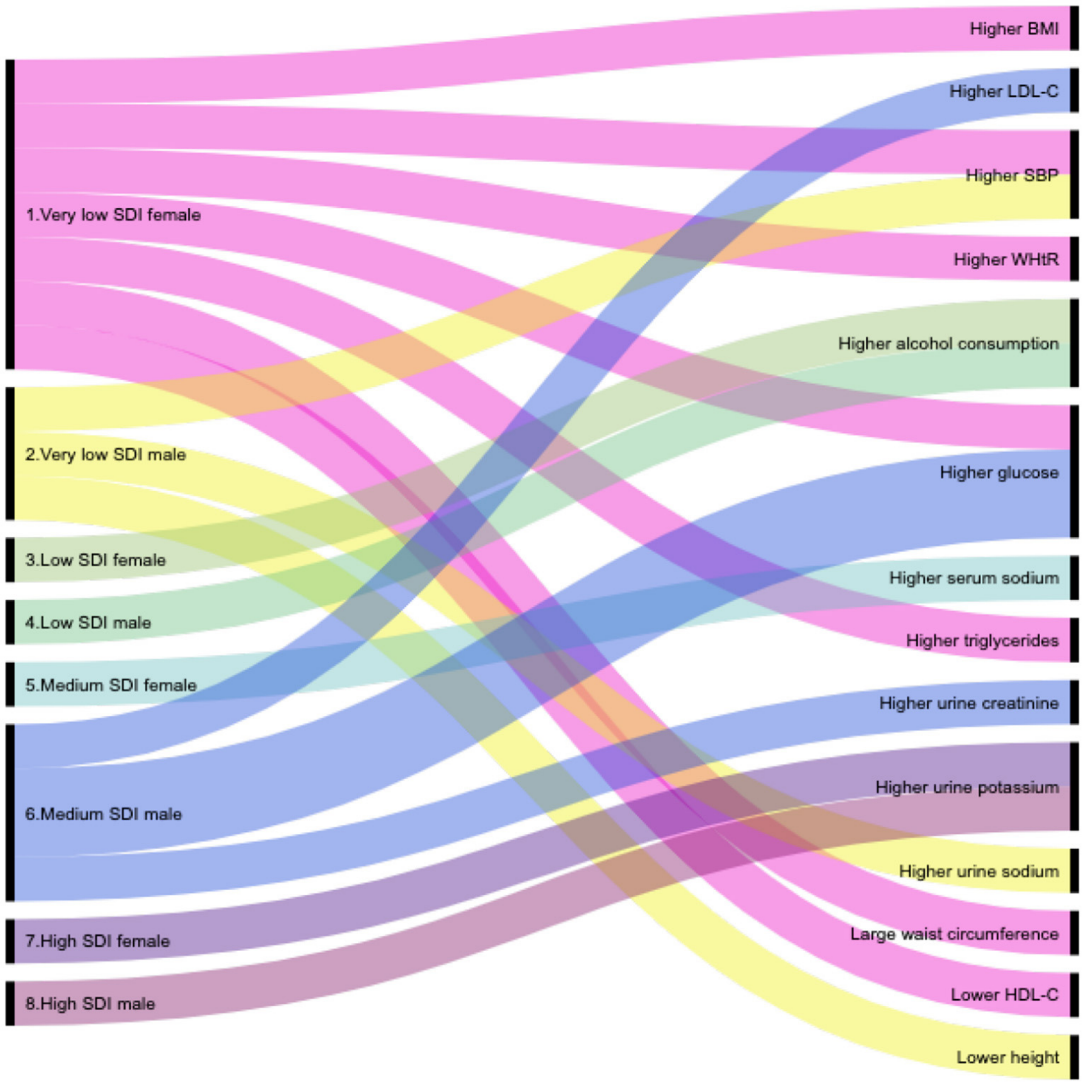
Alluvial diagram of the main cardiovascular risk factors by Social Development Index level.

In Table 4, the distribution of *anthropometric and clinical parameters* among SDI's levels by sex is revealed. These parameters showed to be statistically associated with the SDI's levels in both sexes. This association was however more evident in women. For example, the median of *waist circumference, WHtR, and body mass index (BMI)* tend to decrease as the SDI level increases (from 89 to 85 cm, from 0.581 to 0.535 cm/cm and from 28 to 25 kg/m^2^, respectively) showing that women in disadvantaged social classes were more likely to develop overweight or even obese and accumulate abdominal fat than those in higher SDI's levels (see Figure 2).

**Table 4:** Distribution of anthropometric and clinical parameters among Social Development Index by sex.

					***p*****-Values**	
**Parameters**,	**Very low**	**Low**	**Medium**	**High**	***Kruskal***	***Two-Ways***
**med (IQR 25-75)**					***Wallis***	***ANOVA***
**Age (years)**						
Female	36 (30–45)	40 (31–46)	42 (31–47)	40 (32–45)	*p* < 0.05	0.0069
Male	36 (27–44)	38 (29–44)	39 (30–45)	39 (32–46)	0.1668	
**Weight (kg)**						
Female	66 (59–76)	65 (58–74)	64 (57–73)	63 (56–72)	0.1572	*p* < 0.001
Male	77 (71–85)	78 (69–86)	79 (72-90)	78 (70–85)	0.2851	
**Height (cm)**						
Female	156 (153–160)	157 (153–161)	158 (153–162)	158 (154–163)	*p* < 0.001	*p* < 0.001
Male	169 (165–173)	170 (166–173)	170 (165–174)	171 (166–176)	*p* < 0.05	
**Waist-C (cm)**						
Female	89 (81–96)	87 (80–96)	86 (78–93)	85 (77–92)	*p* < 0.001	*p* < 0.001
Male	94 (88–101)	94 (86–101)	96 (88–104)	93 (87–100)	0.1991	
**WHtR (cm/cm)**						
Female	0.581	0.558	0.547	0.535	*p* < 0.001	*p* < 0.001
	(0.519–0.617)	(0.509–0.610)	(0.494–0.595)	(0.491–0.82)		
Male	0.551	0.555	0.562	0.542	0.1618	
	(0.516–0.601)	(0.507–600)	(0.523–0.606)	(0.506–0.589)		
**BMI (kg/m**^**2**^**)**						
Female	28 (24–31)	26 (24–30)	26 (23–29)	25 (23–29)	*p* < 0.001	*p* < 0.001
Male	27 (24–30)	27 (24–30)	27 (25–31)	26 (24–29)	0.1031	
**SBP (mm Hg)**						
Female	104 (98–111)	105 (97–112)	105 (98–112)	102 (96–110)	*p* < 0.05	*p* < 0.001
Male	113 (105–120)	111 (105–118)	111 (105–119)	109 (103–116)	*p* < 0.05	
**DBP (mm Hg)**						
Female	71 (66–77)	70 (64–76)	70 (63–76)	69 (63–75)	0.0990	*p* < 0.001
Male	75 (71–80)	76 (70–81)	76 (70–81)	74 (69–79)	0.2633	
**HR (beat/min)**						
Female	65.7 (60.7–71.3)	66 (61–71)	67 (61–73)	66 (61–71)	0.4696	*p* < 0.001
Male	63 (59-68)	64 (60-70)	64 (60-70)	63 (60-69)	0.3691	

*Waist-C, waist circumference; WHtR, waist-to-height-ratio; BMI, body mass index; SBP, systolic blood pressure; DBP, diastolic blood pressure*.

Regarding of *SBP* for women and men, again lower values of blood pressure at higher SDI's levels were observed. In women, in the high level: 102 mm Hg, in the medium: 105 mm Hg, in the low: 105 mm Hg, and in the very low: 104 mmHg (*p* < 0.05); and in men, in the high level: 109 mm Hg, in the medium: 111 mm Hg, in the low: 111 mm Hg, and in the very low: 113 mm Hg (*p* < 0.05). However, since this study aims to identify the incidence of hypertension all blood pressure levels (systolic and diastolic) are within normal values—below 120/80 mm Hg (see Table 4).

A similar situation, however, in the opposite direction happened to the *height*. Women and men in higher SDI's levels had larger size than those in lower SDI's levels (see Figure 2); women in high: 158 cm and in very low: 156 cm (*p* < 0.001), and men in high: 171 cm and in very low: 169 cm (*p* < 0.05).

#### Biochemical Test

Distribution of *serum and urinary biochemical tests* among SDI's levels by sex are shown in Table 5, although, biochemical tests ranged within normal values, there were some interesting findings, especially in the case of women, that are worth mentioning. Regarding *glucose* median values, it seems that women in the high SDI level had lower values than those in the other three levels (high: 90 mg/dl vs. medium: 92 mg/dl, low: 92 mg/dl and very low: 92 mg/dl, *p* < 0.001). In relation with *HDL-C*, an increment of the median values was observed as SDI level increased; in very low: 48 mg/dl, in low: 50 mg/dl, in medium: 51 mg/dl, and in high: 53 mg/dl (*p* < 0.001). In the case of *triglycerides*, the opposite pattern was observed; serum median values were lower in higher SDI's levels (high: 104 mg/dl, medium: 107 mg/dl, low: 113 mg/dl, and very low: 119 mg/dl, *p* < 0.001) (see Figure 2).

**Table 5:** Distribution of biochemical test among Social Development Index by sex.

					***p*****-Values**	
**Values**,	**Very low**	**Low**	**Medium**	**High**	***Kruskal***	***Two-Ways***
**med (IQR 25-75)**					***Wallis***	***ANOVA***
**SERUM PARAMETERS**						
**Glucose (mg/dl)**						
Female	92 (87–99)	92 (86–97)	92 (87–97)	90 (85–96)	*p* < 0.001	*p* < 0.001
Male	93 (88–99)	95 (89-99)	95 (90–101)	93 (88–99)	*p* < 0.05	
**Uric acid (mg/dl)**						
Female	4.7 (4.1–5.4)	4.7 (4–5.4)	4.6 (4.1–5.3)	4.6 (4–5.3)	0.4944	*p* < 0.001
Male	6.4 (5.5–7.2)	6.4 (5.6–7.2)	6.5 (5.9–7.4)	6.5 (5.6–7.2)	0.2978	
**Sodium (mmol/l)**						
Female	140 (140–141)	140 (140–141)	141 (140–142)	140 (140–141)	*p* < 0.05	*p* < 0.001
Male	141 (140–142)	141 (140–142)	141 (140–142)	141 (140–142)	0.2776	
**Cholesterol (mg/dl)**						
Female	174 (156–198)	177 (158–199)	181 (161–204)	181 (163–205)	0.1196	0.1873
Male	175 (160–200)	178 (160–208)	188 (163–206)	183 (160–207)	0.3682	
**HDL-C (mg/dl)**						
**(mg/dl)**						
Female	48 (41–55)	50 (42–58)	51 (43–61)	53 (45–62)	*p* < 0.001	*p* < 0.001
Male	42 (36–47)	42 (36–49)	42 (35–47)	42 (36–48)	0.7742	
**LDL-C (mg/dl)**						
Female	110 (98–131)	114 (96–135)	116 (97–137)	116 (98–136)	0.5667	*p* < 0.001
Male	111 (99–130)	120 (97–141)	126 (105–142)	121 (100–141)	0.0543	
**Triglycerides (mg/dl)**						
Female	119 (90–185)	113 (81-156)	107 (83–150)	104 (78–141)	*p* < 0.001	*p* < 0.001
Male	143 (99–228)	146 (95-207)	149 (114–205)	130 (102–195)	0.3843	
**Atherogenic index**						
**(LDL-C/HDL-C)**						
Female	2 (2–3)	2 (2–3)	2 (2–3)	2 (2–3)	0.0550	*p* < 0.001
Male	3 (2–3)	3 (2–4)	3 (3–4)	3 (2–4)	0.1600	
**Creatinine (mg/dl)**						
Female	0.70	0.71	0.71	0.71	0.2616	*p* < 0.001
	(0.62–0.76)	(0.64–0.77)	(0.65–0.78)	(0.64–0.79)		
Male	0.93	0.93	0.94	0.95	0.7347	
	(0.82–1.03)	(0.86–1.02)	(0.85–1.02)	(0.86–1.02)		
**URINARY PARAMETERS**						
**Sodium (mmol/24 h)**						
Female	117 (87–152)	109 (82–141)	115 (85–155)	112 (83–147)	0.1954	*p* < 0.001
Male	163 (120–200)	135 (95–180)	141 (106–194)	144 (107–188)	*p* < 0.05	
**Potassium (mmol/24 h)**						
Female	43 (33–55)	41 (30–52)	44 (32–56)	45 (34–57)	*p* < 0.05	*p* < 0.001
Male	50 (37–60)	46 (35–62)	50 (38–69)	52 (38–65)	*p* < 0.05	
**Creatinine (mg/24 h)**						
Female	936	922	898	928	0.4450	*p* < 0.001
	(758–1,122)	(724–1,078)	(722–1,103)	(773–1,095)		
Male	1,454	1,354	1,479	1,399	*p* < 0.05	
	(1,225–1,671)	(1,107–1,639)	(1185–1,719)	(1,159–1,677)		

*HDL-C, high-density lipoprotein cholesterol; LDL-C, low-density lipoprotein cholesterol*.

In the case of men, significant differences were identified in the median of *glucose* values among SDI's levels without a specific pattern (very low: 93 mg/dl, low: 95 mg/dl, medium: 95 mg/dl, and high: 93 mg/dl, *p* = 0.0335; see Table 5).

Regarding urinary tests also shown in Table 5, which were within normal values, an interesting behavior was also observed. The sodium and potassium median values showed significant differences among SDI's levels, being more evident in men. For instance, sodium median values were significantly larger in men in the lower SDI's levels than in the higher levels (very low: 163 mmol/24 h, low: 135 mmol/24 h, medium: 141 mmol/24 h, and high: 144 mmol/24 h, *p* < 0.05); while potassium showed a different pattern, larger median values were observed in higher SDI's levels than in lower levels (high: 52 mmol/24 h, medium: 50 mmol/24 h, low: 46 mmol/24 h, and very low: 50 mmol/24 h, *p* < 0.05), this pattern was also identified in women (high: 45 mmol/24 h, medium: 44 mmol/24 h, low: 41 mmol/24 h, and very low: 43 mmol/24 h, *p* < 0.05). Furthermore, worth mentioning that sodium levels between sexes were significantly different: men had larger median values than women (see Figure 2).

#### Others Risk Factors

The distribution of *CVRF among SDI's levels* by sex showed some differences regarding alcohol consumption, physical activity, and psychological stress (see Tables 6, 7). In relation with *alcohol consumption*, the proportion of women with this habit in the high level of SDI was significantly larger than those without the habit (27 vs. 20%), however the significance did not hold after Bonferroni's correction, *p* < 0.05 (after Bonferroni's correction, *p* = 0.22). In the case of men, the proportion of those with this habit in the high level of the SDI was significantly larger than those without it (27 vs. 17%), whereas in the very low level of the SDI the proportion of men without the habit was larger than those with the habit (21 vs. 11%, *p* < 0.001, after Bonferroni's correction *p* = 0.004).

**Table 6:** Distribution of cardiovascular risk factors among Social Development Index between female and male.

					***p*****-Values**	
					**χ^2^*or C's V***	**Bonferroni**
**CVRF n(%)**	**Very low**	**Low**	**Medium**	**High**	**Uncorrected**	**Corrected**
**SMOKING HISTORY**						
**Positive**						
Female	97 (13)	285 (38)	176 (24)	183 (25)	0.465	1.86^*a*^
Male	75 (14)	215 (38)	129 (23)	142 (25)	0.929	3.72^*b*^
**Negative**						
Female	92 (16)	213 (37)	126 (22)	142 (25)	0.986	3.94^*c*^
Male	26 (12)	81 (39)	52 (25)	50 (24)	0.565	2.26^*d*^
**ALCOHOL CONSUMPTION**						
**Positive**						
Female	115 (13)	311 (37)	193 (23)	230 (27)	*p* < 0.05	0.22^*a*^
Male	64 (11)	224 (38)	143 (24)	161 (27)	*p* < 0.001	0.004^*b*^
**Negative**						
Female	74 (16)	187 (40)	109 (25)	95 (20)	0.468	1.87^*c*^
Male	37 (21)	72 (41)	38 (21)	31 (17)	0.455	1.82^*d*^
**DRUNKENNESS**						
**Positive**						
Female	11 (15)	28 (37)	20 (26)	17 (22)	0.897	3.59^*a*^
Male	21 (15)	44 (32)	35 (25)	38 (28)	0.369	1.48^*b*^
**Negative**						
Female	178 (14)	470 (38)	282 (23)	308 (25)	0.825	3.30^*c*^
Male	80 (13)	252 (40)	146 (23)	154 (24)	0.716	2.86^*d*^

a *Comparison among women*.

b *Comparison among men*.

c *Comparison between women and men in the first category*.

d *Comparison between women and men in the second category*.

**Table 7:** Distribution of cardiovascular risk factors among Social Development Index between female and male.

					***p*****-Values**	
					**χ^2^*or C's V***	**Bonferroni**
**CVRF n(%)**	**Very low**	**Low**	**Medium**	**High**	**Uncorrected**	**Corrected**
**PHYSICAL ACTIVITY**						
**Low**						
Female	19 (13)	63 (41)	33 (22)	37 (24)	0.866	4.33^*a*^
Male	11 (11)	36 (35)	27 (27)	27 (27)	0.849	4.25^*b*^
**Moderate**						
Female	95 (15)	227 (36)	145 (23)	162 (26)	0.697	3.49^*c*^
Male	46 (13)	135 (38)	84 (23)	94 (26)	0.799	4.00^*d*^
**High**						
Female	75 (14)	208 (39)	124 (23)	126 (24)	0.982	4.91^*e*^
Male	44 (14)	125 (40)	70 (23)	71 (23)		
**PSYCHOLOGICAL STRESS**						
**Low**						
Female	4 (25)	9 (56)	2 (13)	1 (6)	*p* < 0.05	0.26^*a*^
Male	1 (5)	8 (38)	10 (48)	2 (9)	0.104^*^	0.52^*b*^
**Moderate**						
Female	183 (14)	486 (38)	296 (23)	322 (25)	0.434^*^	2.17^*c*^
Male	100 (13)	238 (38)	171 (23)	190 (26)	0.967	4.84^*d*^
**Severe**						
Female	2 (18)	3 (27)	4 (37)	2 (18)	0.674^*^	3.37^*e*^
Male	0	5 (100)	0	0		

a *Comparison among women*.

b *Comparison among men*.

c *Comparison between women and men in the first category*.

d *Comparison between women and men in the second category*.

e *Comparison between women and men in the third category*.

* *p-value of Cramer's V coefficient*.

In relation to the variables related to *physical activity and psychological stress*, the highest proportion of women and men with a moderate degree of both parameters were mainly concentrated in the low, medium, and high SDI's levels. There was no statistical difference between the groups compared (see Table 7).

###  Multivariate Analysis

The multivariate analysis is shown in Table 8. A strong and significant correlation was identified between the dependent variable U1 (sociodemographic variables) and the independent variable V1 (biological variables) (80.38%). Furthermore, the variance of this canonical correlation coefficient (CCC) accounts for the 65% of the differences among and between the categories of each variable within the dependent variable U1. This means that for example regarding the SDI, individuals classified in the very low, low, medium, or high SDI level are different from each other and its correlation with the V1 variables is also different. These results show that levels of these biochemical components and systolic blood pressure, WHtR, and alcohol consumption are strongly determined by sociodemographic conditions such as the SDI, the sex, the marital status, the educational level, and the occupational class. The estimated canonical loadings for the U1 variables showed that educational level and marital status are strongly associated with the CCC (−75.52 and 60.79%, respectively) in opposite directions. This is the case of the V1 variable the WHtR, alcohol consumption and serum glucose levels (75.12, −49.20, and 47.52%, respectively) were the once with the strongest association (see Supplementary Table 1).

**Table 8:** Results of the canonical correlation analysis.

	**Canonical**	**Linear**			**Canonical**
	**Variables**	**coefficients**	***P*-value**	**95% Confidence Interval**	**Coefficient**
U1	SDI	−0.2614326	*p* < 0.001	−0.3833; −0.1396	
	Sex	−0.0099968	0.936	−0.2548; 0.2348	
	Marital Status	0.6774097	*p* < 0.001	0.5056; 0.8493	
	Educational Level	−0.6809158	*p* < 0.001	−0.8238; −0.5381	
	Occupational Class	0.1532161	*p* < 0.001	0.0911; 0.2153	
					0.8038
V1	Glucose	0.0188744	0.005	0.0056; 0.0322	
	Uric acid	−0.1552788	*p* < 0.001	−0.2733; −0.0373	
	HDL-C	−0.0044275	0.456	−0.0161; 0.0072	
	LDL-C	0.0009298	0.655	−0.0032; 0.0050	
	Triglycerides	0.0024978	*p* < 0.001	0.0012; 0.0038	
	Serum creatinine	−0.4224985	0.413	−1.4334; 0.5884	
	Urinary creatinine	0.0007612	*p* < 0.001	0.0003; 0.0012	
	Urinary potassium	−0.0226786	*p* < 0.001	−0.0306; −0.0148	
	Systolic blood pressure	0.0124834	0.04	0.0006; 0.0244	
	WHtR	7.211526	*p* < 0.001	5.2514; 9.1717	
	Alcohol consumption	−0.9411592	*p* < 0.001	−1.1991; −0.6833	

*P-value for Wilks' λ= p < 0.001. SDI, Social Development Index; WHtR, waist-to-height ratio; HDL-C, high-density lipoprotein cholesterol; LDL-C, low-density lipoprotein cholesterol. Reference values: SDI = very low level; Sex = male; Marital status = single; Educational level = Elementary school; Occupational class = Student*.

We can see that lower levels of SDI, along with being a man, being married, having a lower educational level, and being unemployed were correlated with several known CVRF such as serum *glucose, uric acid, HDL-C, LDL-C, triglyceride, and creatinine*, also urinary *creatinine and potassium* as well as *SBP, WHtR, and alcohol consumption*.

## Discussion

Until recently, science has relied on the biological aspects of diseases to try to explain the causality of many chronic degenerative diseases, however failing to fully explain it. Nowadays, knowledge goes beyond the physiological aspects into the complex interaction between social and economic conditions that have also reveled a role in the development of these health conditions (26, 57, 58).

The present study showed that the SDI can be a standardized measure to explore socioeconomic status within developing countries, where inequalities are high, and where the relationship with risk factors is still little studied. The rationale behind studying the association between SDI and CVRF instead of actual disease or co-morbidities is that by unveiling such links in healthy individuals with known risks, one may be able to derive knowledge that could be translated into primary prevention strategies. Public health policy may be designed with a view to early social interventions in neighborhoods with increased risk associated features (see Supplementary Note 1) (9, 59, 60).

One of the first findings was a persistent social lag for both, men and women, in *quality and available space in the home and access to social security* in the lower strata of the SDI. However, in relation with *educational access and durable goods*, most of them were at the medium or high level of the SDI. This panorama is important in order to contextualize the health burden of CVRF impose to the most vulnerable population if the social and economical disparities continue to increase, particularly in relation to health care access, a major challenge that the Mexican health system is facing (see Supplementary Note 2) (61–63).

Regarding anthropometric and clinical parameters, we identified that women had larger values of waist circumference, WHtR, and BMI in lower levels of the SDI. SBP also was significantly higher in lower SDI's levels in men and in women as well. In relation to biochemical tests, an association with SDI's levels in women was observed. Larger values serum glucose and triglycerides, and lower values of HDL-C were related to lower levels of SDI. In the case of urine tests, this same pattern was found in relation to sodium levels in men, whereas for potassium, both sexes exhibit an inverse pattern.

Several studies have identified associations between CVRF and SES. People in socioeconomic disadvantage have a higher likelihood of presenting CVRF, than those in socioeconomic wellness. In a review by Zhu et al., unhealthy nutrition patterns were more frequent in lower SES (26). This SES disadvantage seems to be gender related. Studies in middle-income countries have found that women in lower SES have a larger prevalence of overweight or obesity and smoking habit than men, whereas the risk of stroke associated with increments in weight can be higher in men than in women (2). In a survey of middle-income countries by Bovet et al. (3), 68.3% of women and 52% of men had overweight and 35% of women and 15% of men were obese. It has been reported that in middle-income countries, men smokes 5 times more than women (48 vs. 10%), whereas women who smoke might have a greater relative risk of CVD (2, 64, 65). Another study in a middle-income country showed that females suffer more often from social and early-life economic disadvantage conditions as being less educated, economically inactive, poor, and with middle wealth index (66); these situations make women more prone to develop CVRF (67).

Our results are also comparable to other Mexican studies such as the *Lindavista Study*, a project for multiple intervention trial on cardiovascular risk factors (68) and the *PRIT study* (Prevalence of Cardiovascular Risk Factors in Hospital General de Mexico Workers) (69), where 70% of participants came from urban middle-class population and had high school or higher education, whereas in our study, all participants came from an urban population however from a mixture of social classes. Furthermore, their educational level was high school or higher in 84.51%. Regarding anthropometric and clinical parameters, approximately 75% of the subjects were BMI ≥ 25 kg/m^2^; 88% of the women had an abdominal circumference ≥ 80 cm, while 74% of the men had a ≥ 90 cm; these results are also similar to ours. Median BMI was ≥ than 25 kg/m^2^ for the very low, the low, and the medium SDI's levels, and the median waist circumference was around ≥ 80 and 90 cm for women and men, respectively, in all SDI's levels. Also, the study reveled HDL-C level in women has been often higher than in men. The opposite tendency happened in the case of triglycerides as well as the less common smoking history in men, and the same tendency was observed in our study (68).

Additionally, we found that a lower level of SDI, lower educational levels, being unemployed, and single were associated with increased serum glucose, LDL-C, triglycerides, urinary creatinine, systolic blood pressure and WHtR, as well as, a decreased serum levels of uric acid, creatinine, urinary potassium, and lower alcohol consumption. Aside from these broad context issues, our analysis was also able to unveil the associations between particular cardiometabolic factors and the socioeconomic development, approximated by SDI but also by educational level and occupational class. This interaction is indeed a nascent research area in public health that is starting to gain attention recently (see Supplementary Note 3) (70–72).

Overweight and obesity were also found to be positively correlated with human development index and urban environments in India (72) as well as with female literacy, an indirect indicator of overall human development in emerging economies. The development of hypercholesterolemia, hypertriglyceridemia, and hypoalphalipoproteinemia is associated with the presence of obesity and its determinants related to lifestyle. These CVRF are the most prevalent in Mexico and have been reported to be even higher than in some of the similar countries (see Supplementary Note 4) (63).

Likewise, another review study, by Mendoza-Herrera et al. (63), on the attributable burden of cardiovascular diseases and risk factors focused mainly on the prevalence of dyslipidemia in mega-countries with different development indices (low, Nigeria; medium, India; high, such as China, Brazil, and Mexico and very high: the United States and Japan) revealed that after obesity, hypoalphalipoproteinemia (HDL-C <50 mg/dl for women and <40 mg/dl for men) and high LDL-C (≥100 mg/dl) were the most prevalent CVRF in Mexican adults since reported in 2006. Also the study revealed that the most recent prevalence reports of hypertriglyceridemia and hypercholesterolemia in Mexico were higher compared with that in India, Nigeria, China, Japan, and in the United States. A larger prevalence of high LDL-C was found in Mexico (46.0%, LDL-C ≥130 mg/dl) only after Brazil [57.6% (women) 58.5% (men)] (63).

In summary, in this study, we demonstrated that Mexican urban population accumulates several important risk factors related to metabolic abnormalities (higher serum glucose, LDL-C, HDL-C, triglycerides, uric acid, creatinine as well as urinary potassium and creatinine, systolic blood pressure, and WHtR). A large percentage of the population studied in lower levels of development is deprived of access to health care facilities. The problem seem to be more complex for women, in whom low levels of access to formal employment and in consequence limited access to social protection persist. Also the analysis shows how particular SDI components (access to housing and health care) deficits could reduce the capacity to change that panorama (see Figure 2).

Mexico and nations with a similar or lower HDI experience greater challenges related to CVRF (73). In these countries, dietary risk factors contribute more to cardiovascular mortality. In addition, there is a greater probability of premature death from CVD (74). Likewise, both, Mendoza-Herrera et al. (63) and Story et al. (75) stated that in terms of environmental and health policy interventions may be among the most effective strategies for creating population-wide improvements in eating, by modifying the food system, on the one hand: motivating the reduction of high sodium intake, the consumption of sugary drinks, the reduction of tobacco consumption, and promoting active lifestyles. On the other hand, the imposition of taxes on unhealthy foods and monitoring of CVD risk in epidemiological surveillance systems are also promising strategies to reduce CVRF (63, 75). If we do not influence in these identified CVRF as well as in social drivers of health, we run the risk of increasing the burden of chronic degenerative diseases and, at the same time, slowing down social and economic development (76, 77).

###  Scope and Limitations

This study explores the relationship between SDI, as a social determinant of health proxy, and cardiovascular risk within an upper middle income country to analyze how these social inequities in health might be implicated in the development of biological conditions such as the increment in the body mass or in the levels of blood sugar, triglycerides, LDL-C, etc. As a result, some disparities regarding cardiovascular risk factors were observed in lower vs. higher SDI, particularly in women. These findings could facilitate cardiovascular risk factors control planning in countries experiencing both socioeconomic and epidemiological change related to social-economical disparities such as Mexico. This study also has some limitations. Since recruitment was made through massive disseminating methods, participation was voluntary, and women tended to care more for their health and participate more than men, resulting in a slightly biased toward female participants. Another limitation is that we used the SDI data, which are published by the Government of Mexico City and therefore we have to rely on the quality of data compiled by that secondary source.

## Conclusion

The present study shows how SDI and other human development components impact directly and indirectly the CVRF through intermediate issues related to some metabolic aspects, lipids in particular and healthy weight. Concerning sex, in our study population the proportion of young men under 50 reported more traditional risk factors than women in similar age brackets, but when we explored the phenomena by strata of the SDI, the situation seems to turn less favored toward women with low levels of development.

We found SDI, but also two of its components: *Quality and available space in the home* and *Health care access* as elements of general concern for public health interventions and nutritional conditions, which may help reduce health inequalities in the Mexican population, in particular in large metropolitan areas such as Mexico City. So, we presented SDI as a feasible and a complementary instrument to collect information about socioeconomic strata, Mexican neighborhoods, and their relation to cardiovascular health at the population level with public health policy design in mind.

We hope that this study will help to conduct future research using a socially oriented perspective in order to implement primary prevention and health promotion strategies in scheme of cardiovascular risk factors and to continue monitoring it in future studies. As discussed previously, the careful analysis of population level association of human development components and risk factors for cardiovascular disease may provide health policy makers and urban setting decision makers with solid tools for scientifically guided intervention designs.

## Data Availability Statement

The original contributions generated for the study are included in the article/Supplementary Material, further inquiries can be directed to the corresponding author/s.

## Ethics Statement

This study was approved by the Research and Ethics Committees at INC-ICh with number 13-802.

## Author Contributions

MM-G, GG-E, MV, and EH-L have been involved in conceptualization, methodology, formal analysis, and data interpretation. GG-E, JR-G, DM-P, CM-R, and MV have been involved in data collection, data curation, and management. MM-G, GG-E, MV, and EH-L have been involved in the preparation of the manuscript. EH-L and MV have been involved in funding acquisition. All authors have read and agreed to the published version of the manuscript.

## Conflict of Interest

The authors declare that the research was conducted in the absence of any commercial or financial relationships that could be construed as a potential conflict of interest.
