# Supplementary material for: Cardiovascular Risk Factors and Social Development Index
Source: Front Cardiovasc Med. 2021 Feb 23;8:631747. doi: 10.3389/fcvm.2021.631747 (PMC7940205; doi:10.3389/fcvm.2021.631747)
Supplement: Supplementary file 1 [file Data_Sheet_1.PDF]

Cardiovascular Risk Factors and Social Development Index

DESCRIPTION OF THE PROSPECTIVE LONGITUDINAL STUDY OF RISK FACTORS FOR HYPERTENSION INCIDENCE IN A MEXICO CITY POPULATION: THE TLALPAN 2020 COHORT

The National Institute of Cardiology Ignacio Chavez (INCICh) is a public hospital flagship specialized institution for the treatment of cardiovascular diseases in Mexico (Cruz-Ávila et al., 2020). The Tlalpan 2020 cohort is an observational, longitudinal, prospective study that is conducted in Mexico City, Mexico. This study was approved by the Research Ethics Board for Biomedical Research in humans of INCICh under number 13-802 (Colín-Ramírez et al., 2017).

The enrollment started in September 2014 and is ongoing. Healthy volunteers are women and men residents of Mexico City, between 20 and 50 years old. No participants used anti-hypertensive therapy, since none of them suffer hypertension. Individuals previously diagnosed with some disease like diabetes mellitus, dysthyroidism, cerebrovascular disease, ischaemic cardiomyopathy, acute coronary syndrome, cancer with an effect on survival, pregnant women and those taking medications that have an effect on blood pressure, also person with cognitive and mental disabilities are excluded. All volunteer gave written informed consent to participate in this study.

For recruitment of the general population were employed: Massive dissemination methods, such as flyers/posters (delivered in health, community, work, cultural centres and so on), social media tools (Facebook and Twitter), communication media (radio and television), newspaper, magazine advertisements and personal referrals.

Clinical assessments, biologic samples and manage data related to this study are conducted at the INCICh. The participants have been evaluated every 2 years over a period of 10 years or until they develop HTN (primary outcome variable). During the initial visit (baseline), clinical, anthropometric, biochemical, diet, physical activity, stress, sleep quality, sociodemographic data, personal and family pathological antecedents, and alcohol and tobacco consumption are collected. All instruments will be applied by personnel trained.

This is the first cohort in Mexico aimed to study the impact of traditional and non-traditional risk factors for systemic hypertension. In this case, with one of the less explored social determinants of health (the Index of Social Development). Participants are contacted every 6 months via email to thank them for their participation, keep them informed about its progress and update their contact information. Additionally, participants are contacted by telephone annually to verify whether they have been diagnosed with HTN and to corroborate their contact information again.

Evaluations and instrumentation

Anthropometry and clinical parameters

Blood pressure will be measured in the left arm three times with a 3 min interval between each measurement, with the patient remaining in a sitting position for at least 10 min before the first evaluation. If one of the three measurements is quite different, a four measure will is taken. The value recorded is the average of the three closer measurements. The hypertension is defined as a systolic blood pressure $\geq$

140mm Hg and/or a diastolic blood pressure $\geq$ 90mm Hg (Chobanian et al., 2003). A cuff in accord with the diameter of the arm and a mercury sphygmomanometer, previously calibrated in the INCICh, is used by the assessment. Heart rate (beats per minute) and respiratory rate (breaths per minute) are also measured.

Evaluation of anthropometric measurements (weight, height and waist circumference) are performed with the patient fasting, shoeless and wearing a hospital gown in accordance with the procedures described by 'The International Society for the Advancement of Kinanthropometry (ISAK). A mechanical column scale (SECA 700) with a capacity of 220 kg and precision of 0.05 kg is used and the weight is recorded to the nearest 100 g. Height will be measured with a stadiometer SECA 220. Waist circumference is measured at the level of the narrowest point between the lower costal border and the iliac crest by using a measuring tape made of glass fibre BodyFlex, with a length of 150 cm and precision of 1mm. Body Mass Index (BMI) was categorized based on World Health Organization definitions (normal, overweight, or obesity) (Organization, 2000).

Biological test

The biochemical samples were measured in automatic analyzers at the Central Laboratory of INCICh. Blood samples were obtained after an overnight fast of 12 hours. Fasting plasma glucose (70-105 mg/dl), triglycerides (40-200 mg/dl), low density lipoprotein cholesterol (LDL-C) (80-130 mg/dl), high-density lipoprotein cholesterol (HDL-C) (women: $>$ 50 mg/dl and men: $>$ 40 mg/dl), total cholesterol (140-200 mg/dl), uric acid (women: 3.80-6.20 mg/dl and men: 4.80-8.00 mg/dl), serum creatinine (women: 0.60-1.00 mg/dl and men: 0.70-1.30 mg/dl), atherogenic Index (LDL/HDL, elevated defined as a value $>$ 4) and serum sodium, (136.00-145.00 mmol/l). Altered lipid profile is defined according to the Adult Treatment Panel III criteria: high total cholesterol when $>$5.2mmol/L, low HDL-C when $<$1.0mmol/L for men and $<$1.3mmol/L for women, high LDL-C when $>$3.4mmol/L and high triglycerides when 1.7mmol/L.

Urine sample data in 24 hours (24 h) were also obtained. For a correct urine collection, the participant was given precise and clear indications, several days in advance of their appointment (discard the first urine in the morning and collect all urine for a period of 24 hours, including the first urine of the following morning, which will be the day of the appointment). Urinary sodium and potassium are determined by the ion selective electrode method, and urinary creatinine is determined by Jaffe's colorimetric assay using an automated analyser. The urine sample is considered complete when urinary creatinine levels are within the standard creatinine excretion rate (133–221 μmol/ kg/24hours for men and 88–177 μmol/kg/24hours for women) (Wielgosz et al., 2016). The reference values of urinary variables are following: for creatinine in women between 740-1570 mg/24 h and for men between 1040-2350 mg/24 h, for sodium between 40.00-220.00 mmol/24 h and for potassium excretion between 25.00-125.00 mmol/24 h. Sodium and potassium excretion was reported in mmol/24 h (or equivalently mEq/24 h).

Others risk factors

Also, antecedents of alcohol and tobacco frequency of consumption are collected (daily, every other day, every weekend, every 2 weeks, once a month or less than once a month). Participants who report smoking at least 100 cigarettes in their lifetime and who, at the time of the survey, smoked either every day or some days would be classified as current smokers (Malarcher et al., 2009). The physical activity antecedent (as measured by the long version of *International Physical Activity Questionnaire, IPAQ: categorized into low, moderate, or high physical activity levels* (Craig et al., 2003)) and psychological stress level (as determined by the *State-Trait Anxiety Inventory, STAI* Spanish version; categorized into low, moderate, or severe psychological stress (Spielberger, 2013)) are obtained. Also, Sleep disorders are evaluated by the Spanish-language Medical Outcomes Study-Sleep scale of 12 items to assess the last week of sleep. Dietary

intake is evaluated using a semiquantitative questionnaire validated in a Mexican population developed by the National Institute of Public Health of Mexico (Hernández-Avila et al., 1998).

Sociodemographic data

Through a personal interview, the following demographic and socioeconomic data were obtained: Marital status (single, married and other), educational level concluded (elementary school, junior high school, higher and postgraduate), occupational class (student, business executive, housekeeper, professional, manually qualifies, manually unqualified, other and unemployed). Also, SDI strata was calculated from the housing, geographic location within the 16 boroughs of CDMX self-reported by the participants.

Final considaration

After a pilot study was carried out, reorganisation into four steps was implemented: 1) reception of participants, 2) collection of biological samples, 3) clinical and anthropometric measurements and 4) face-to-face interviews. The approximate time to complete the visit was estimated at 2 hours per participant.

Regarding data protection, each of the participants was assigned a unique identification number in the study. All access to the information is managed by rigorous security controls, in accordance with the Official Mexican Standard NOM-024-SSA3-2012, ‘Electronic record Information systems for health.

Table S1. Results of the canonical correlation coefficientes

Canonical variables	Linear coefficientes
**Canonical loadings for variable list U1**	
SDI	-0.4815
Sex	-0.0289
Marital Status	0.6079
Educational Level	-0.7552
Occupational Class	0.4668
**Canonical loadings for variable list V1**	
Glucose	0.4752
Uric acid	0.1366
HDL-C	-0.3679
LDL-C	0.1426
Triglycerides	0.4190
Serum creatinine	-0.0861
Urinary creatinine	0.0890
Urinary potassium	-0.2526
Systolic blood pressure	0.3929
Waist-to-Height-Ratio	0.7512
Alcohol consumption	-0.4920

SDI, Social Development Index; *WHtR*, Waist-to-Height-Ratio; *HDL-C*, High-density lipoprotein cholesterol; *LDL-C*, Low-density lipoprotein cholesterol. Reference values: *SDI*=very low level; *Sex*=male; *Marital status*= single; *Educational level*=Elementary school and *Occupational class*=Student.

CANONICAL CORRELATION COEFFICIENTES

ADITIONAL NOTES

In this study, we analyzed the association between CVRF and SDI in the Mexican cohort study Tlalpan 2020. The multivariate analysis showed there were significant and positive correlations between SDI and several CVRF.

1. Public health policy may be designed with a view to early social interventions in neighborhoods with increased risk associated features, e.g. by recreation environments, active transportation, web and mobile-based interventions or implementing early onset nutrition educative measures in disadvantaged populations (Sallis et al., 2012; Gittelsohn and Trude, 2017; Kankanhalli et al., 2019).
2. At the same time, there are many possibilities to change some attitudes, to improve lifestyles based on the fact that people with greater access to education, may have the advantage and the possibilities to attain awareness and self-control of the main CVRF, such as alcohol consumption, physical activity, smoking, and eating habits (Redondo et al., 2011).
3. Low values of education, low levels of occupational class and low levels of SDI are related to increased weight may be non-surprising since the metropolitan context in Mexico is related to a sedentary

lifestyle. Our results are in actual agreement with those recently found in similar urban settlements in a Latin American context (Brazil) (Vale et al., 2019).

4. For example, in Mexico and India, overweight adults are 5.25 and 4.15 times more likely, respectively, to have mixed dyslipidemia and hypertriglyceridemia (Mendoza-Herrera et al., 2019).
5. Both, the general trends discussed in the previous lines and the particular cases just considered point out the relevance of studying – aside from the social determinants of health at the individual level – population level metrics such as the ones captured by human development components such as SDI and its component. These metrics may ease clearer schemes for the implementation of health policy at the population level whose effects on public health are advisable as more broad. However, there is still a lack of solidly founded quantitative evidence of these phenomena, hence more studies of human development components and health risks are needed, in particular for metropolitan urban settings.

REFERENCES

Cruz-Ávila HA, Vallejo M, Martínez-García M, Hernández-Lemus E. Comorbidity networks in cardiovascular diseases. *Frontiers in physiology* **11** (2020) 1009.

Colín-Ramírez E, Rivera-Mancía S, Infante-Vázquez O, Cartas-Rosado R, Vargas-Barrón J, Madero M, et al. Protocol for a prospective longitudinal study of risk factors for hypertension incidence in a mexico city population: the tlalpan 2020 cohort. *BMJ open* **7** (2017) e016773. doi:10.1136/bmjopen-2017-016773.

Chobanian AV, Bakris GL, Black HR, Cushman WC, Green LA, Izzo Jr JL, et al. Seventh report of the joint national committee on prevention, detection, evaluation, and treatment of high blood pressure. *Hypertension* **42** (2003) 1206–1252. doi:10.1161/01.HYP.0000107251.49515.c2.

Organization WH. *Obesity: preventing and managing the global epidemic*. 894 (World Health Organization) (2000).

Wielgosz A, Robinson C, Mao Y, Jiang Y, Campbell NR, Muthuri S, et al. The impact of using different methods to assess completeness of 24-hour urine collection on estimating dietary sodium. *The Journal of Clinical Hypertension* **18** (2016) 581–584.

Malarcher A, Shah N, Tynan M, Maurice E, Rock V, et al. State-specific secondhand smoke exposure and current cigarette smoking among adults-united states, 2008. *Morbidity and Mortality Weekly Report* **58** (2009) 1232–1235.

Craig CL, Marshall AL, Sjöström M, Bauman AE, Booth ML, Ainsworth BE, et al. International physical activity questionnaire: 12-country reliability and validity. *Med Sci Sports Exerc* **35** (2003) 1381–1395. doi:Internationalphysicalactivityquestionnaire:12-countryreliabilityandvalidity.

Spielberger CD. *Anxiety: Current trends in theory and research* (Elsevier) (2013).

Hernández-Avila M, Romieu I, Parra S, Hernández-Avila J, Madrigal H, Willett W. Validity and reproducibility of a food frequency questionnaire to assess dietary intake of women living in mexico city. *Salud publica de Mexico* **40** (1998) 133–140.

Sallis JF, Floyd MF, Rodríguez DA, Saelens BE. Role of built environments in physical activity, obesity, and cardiovascular disease. *Circulation* **125** (2012) 729–737. doi:10.1161/CIRCULATIONAHA.110.969022.

Gittelsohn J, Trude A. Diabetes and obesity prevention: changing the food environment in low-income settings. *Nutr Rev* **75** (2017) 62–69. doi:10.1093/nutrit/nuw038.

Kankanhalli A, Shin J, Oh H. Mobile-based interventions for dietary behavior change and health outcomes: scoping review. *JMIR Mhealth Uhealth* **7** (2019) e11312. doi:10.2196/11312.

Redondo A, Benach J, Subirana I, Martinez JM, Muñoz MA, Masiá R, et al. Trends in the prevalence, awareness, treatment, and control of cardiovascular risk factors across educational level in the 1995–2005 period. *Ann Epidemiol* **21** (2011) 555–563. doi:10.1016/j.annepidem.2011.02.008.

Vale D, Morais CMMd, Pedrosa LdFC, Ferreira MÂF, Oliveira ÂGRdC, Lyra CdO. Spatial correlation between excess weight, purchase of ultra-processed foods, and human development in brazil. *Cien Saude Colet* **24** (2019) 983–996. doi:10.1590/1413-81232018243.35182016.

Mendoza-Herrera K, Pedroza-Tobías A, Hernández-Alcaraz C, Ávila-Burgos L, Aguilar-Salinas CA, Barquera S. Attributable burden and expenditure of cardiovascular diseases and associated risk factors in mexico and other selected mega-countries. *Int J Environ Res Public Health* **16** (2019) 4041. doi:10.3390/ijerph16204041.
